# The Pan African Medical Journal in 2019 - a year in review

**DOI:** 10.11604/pamj.2020.35.99.21683

**Published:** 2020-04-06

**Authors:** Raoul Kamadjeu

**Affiliations:** 1 The Pan African Medical Journal, 3^rd^ Floor, Park Suite Building, Parkland Road, Nairobi, PO.BOX: 38583-00100, Kenya

**Keywords:** PAMJ, research, publication, open access

## Abstract

Since its inception in 2008, the Pan African Medical Journal (PAMJ) enjoyed an exponential growth, not only in the number and geographical diversity of submissions received, but also in the range of services offered to researchers and professionals across the broad spectrum of biomedical and public health sciences. PAMJ in 2019 is: 2705 manuscripts submitted, a cumulative number of 13874 authors from 85 countries, 994 manuscripts accepted for publication and 774 articles published. The PAMJ in 2019 is also an editorial board of 15 and 46 dedicated reviewers to whom we extend our sincere appreciation.

## Editorial

The Pan African Medical Journal (PAMJ) was created in 2008 to expand the publication options of academics, researchers and health professionals in Africa and elsewhere. From a simple debut, with only 2 staff in the early years of its inception, the PAMJ emerges in 2019 as the most important African native biomedical sciences publisher on the continent. Since 2019, the PAMJ portfolio includes two new journals (PAMJ-Clinical Medicine and the PAMJ-One Health) in addition to its existing range of services available to academics and health care professionals. 2019 also saw the launch of the new PAMJ Manuscript Submission System (The PAMJ-Manuscript Hut) and the PAMJ Workflow, the new PAMJ Manuscripts Management Platform.

### Submissions summary

**Figure 1:**
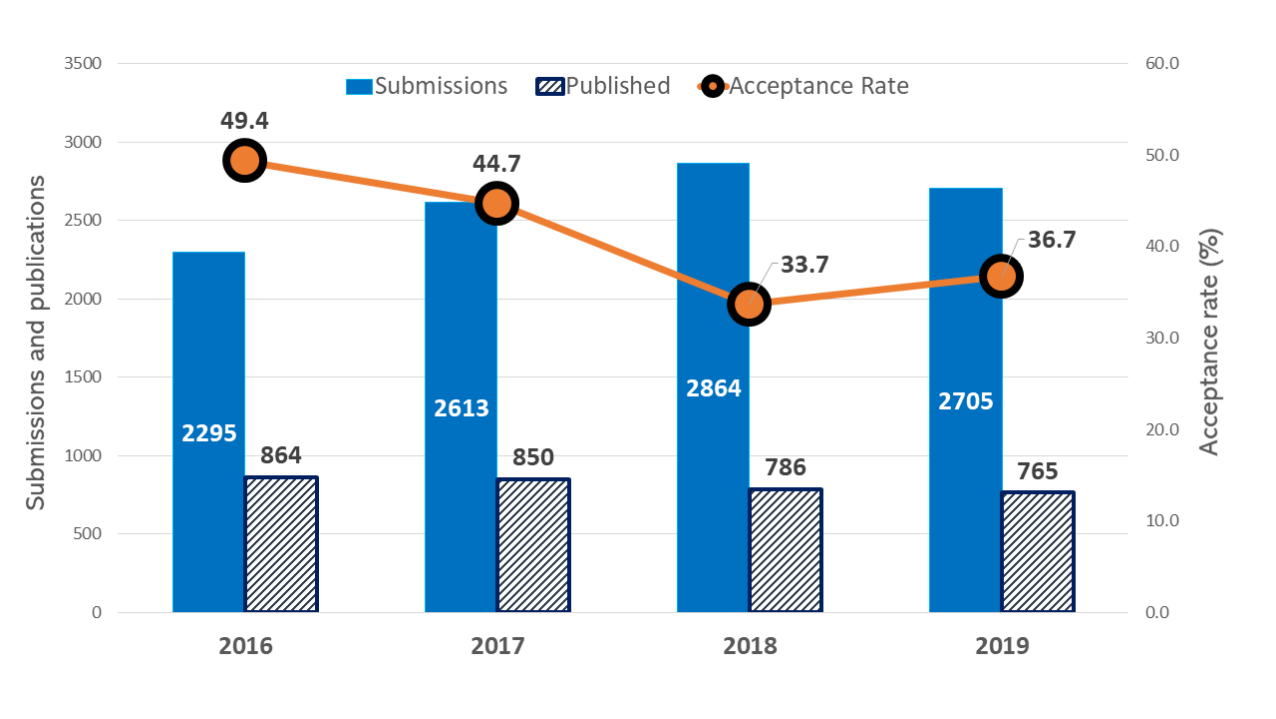
The Pan African Medical Journal-manuscript submissions, publication and acceptance rates (2016-2019)

**Figure 2:**
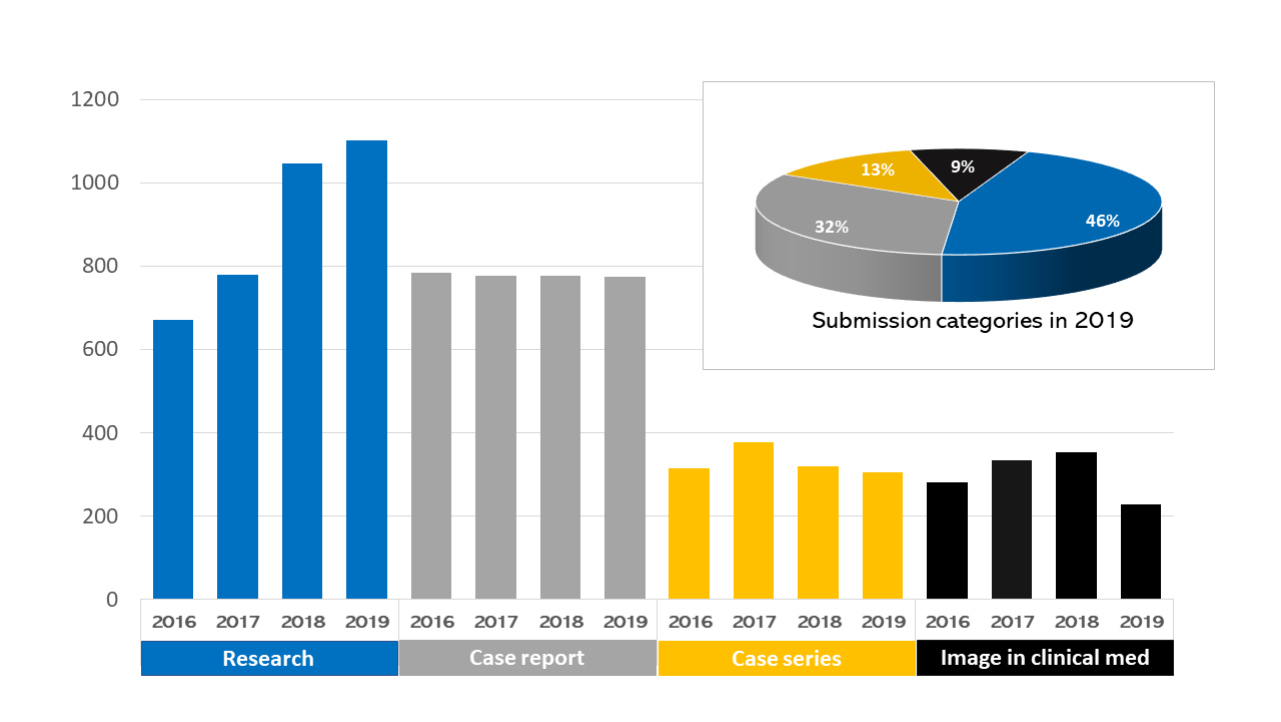
The Pan African Medical Journal-submissions categories (2016-2019)

Overall, 2705 manuscripts with a cumulative number of 13874 authors from 85 countries were submitted to the PAMJ in 2019; 994 of these submissions were accepted for publication the same year (acceptance rate 36.7%) (Figure 1). Notable is the geographical expansion of the countries of origin (assigned by submitting author) of manuscripts submitted to PAMJ, this expansion clearly demonstrates the stature of the PAMJ as an international journal (Annex 1). Three categories dominated submissions to the PAMJ in 2019: research, case reports, case series represented 46%, 32% and 13% respectively, of the total submissions for 2019. Overall, the change in submissions over the last four years continued to be driven by the yearly increase of submissions in the research category (Figure 2). The PAMJ in 2019 was also an editorial board of 15 and 46 dedicated reviewers (Table 1) to whom we extend our sincere appreciation.

**Annex 1:** Country submissions to the PAMJ journals in 2019. Countries are ordered by decreasing shares of research submissions

		Research	Case report	Case series	Images in Clinical medicine	Others	Total
1	Nigeria	166	16	4	0	33	219
2	Ethiopia	137	2	0	0	14	153
3	Cameroon	108	7	4	2	17	138
4	Morocco	84	401	132	155	54	826
5	Tunisia	46	127	61	28	19	281
6	Congo DRC	45	25	6	0	7	83
7	Indonesia	42	1	0	0	4	47
8	Kenya	42	15	1	3	15	76
9	Burkina Faso	38	7	11	0	6	62
10	Ghana	35	4	0	0	8	47
11	Turkey	25	5	2	0	2	34
12	Zambia	24	1	0	0	4	29
13	South Africa	23	5	0	0	3	31
14	Saudi Arabia	21	5	0	0	3	29
15	Uganda	18	1	0	0	0	19
16	Senegal	17	31	17	2	10	77
17	Togo	14	4	5	1	2	26
18	Cote D'Ivoire	14	7	8	0	3	26
19	Iran	13	1	0	0	3	17
20	Tanzania	13	0	0	0	2	15
21	Congo	12	7	0	1	4	24
22	Sudan	12	0	0	0	2	14
23	Benin	10	5	4	0	0	19
24	India	10	4	1	6	3	24
25	Madagascar	10	8	8	3	3	32
26	Rwanda	10	0	0	0	0	10
27	Algeria	9	4	4	0	3	20
28	Guinea	9	0	9	2	2	22
29	Zimbabwe	9	2	0	0	2	13
30	Egypt	8	3	0	0	1	12
31	Mali	7	13	8	6	2	36
32	Mauritania	5	12	6	4	3	30
33	United States	5	3	0	2	8	18
34	Libyan	4	0	0	0	0	4
35	Pakistan	4	1	0	0	0	5
36	China	3	3	0	0	2	8
37	Comoros	3	0	0	0	0	3
38	Gabon	3	0	2	0	3	8
39	Lebanon	3	1	0	0	1	5
40	Burundi	2	1	1	0	0	4
41	Eritrea	2	0	0	0	1	3
42	Greece	2	5	2	3	5	17
43	Iraq	2	0	0	0	0	2
44	Jordan	2	1	0	0	8	11
45	Malawi	2	0	0	0	1	3
46	Mozambique	2	0	0	0	3	5
47	Niger	2	4	2	0	0	8
48	Sierra Leone	2	0	0	0	0	2
49	UAE	2	0	0	1	0	3
50	Antigua And Barbuda	1	0	0	0	1	2
51	Bangladesh	1	0	0	0	0	1
52	Botswana	1	0	0	0	0	1
53	Canada	1	0	0	0	0	1
54	France	1	13	2	0	1	17
55	Guinea-Bissau	1	0	0	0	0	1
56	Republic of Korea	1	0	0	0	0	1
57	Lesotho	1	0	0	0	1	2
58	Liberia	1	0	0	0	1	2
59	Malaysia	1	3	0	1	0	5
60	Namibia	1	0	0	0	1	2
61	Russia	1	0	0	0	0	1
62	Sweden	1	0	0	0	0	1
63	Thailand	1	0	0	0	0	1
64	Afghanistan	0	0	0	0	2	2
65	Albania	0	0	0	0	1	1
66	Angola	0	0	0	0	2	2
67	Argentina	0	0	0	0	1	1
68	Australia	0	0	0	0	2	2
69	Austria	0	1	0	0	0	1
70	Belgium	0	1	0	0	0	1
71	Brazil	0	0	1	1	0	2
72	Central African Republic	0	0	0	0	1	1
73	Chad	0	1	0	0	0	1
74	Chile	0	0	0	0	1	1
75	Djibouti	0	0	1	0	0	1
76	Germany	0	0	0	0	2	2
77	Guadeloupe	0	3	0	0	0	3
78	Italy	0	4	0	2	6	12
79	Kazakhstan	0	0	0	0	1	1
80	Myanmar	0	1	0	0	0	1
81	Oman	0	0	0	0	1	1
82	Portugal	0	1	0	3	0	4
83	Qatar	0	1	0	0	2	3
84	Romania	0	1	0	0	0	1
85	United Kingdom	0	0	0	1	1	2

**Table 1:** The Pan African Medical Journal 2019 reviewers list

**Ajayi Anthony Idowu,** South Africa	**Ajumobi Olufemi,** Nigeria
**Alagbe Adekunle,** Nigeria	**Amponsah Seth,** Ghana
**Banakar M,** Iran	**Belisario Andre Rolim,** Brazil
**Ben Hammamia Mohamed,** Tunisia	**Bigna Jean Joel,** Cameroon
**Chakma Tapas,** India	**Claude Nkfusai Ngwayu,** Cameroon
**Dahmani Fatima,** Morocco	**Danny Kasongo Kakupa,** Congo DRC
**Diallo Alpha,** Guinea	**Elelu Nusirat,** Nigeria
**Fátima Roso Bas,** Spain	**Fondjo Linda Ahenkorah,** Ghana
**Franky Baonga,** Cameroon	**Hernandez Julie H,** United States
**Jemel Manel,** Tunisia	**Joseph Fokam,** Cameroon
**Kaambo Evelyn,** South Africa	**Kamadjeu Martial,** Kenya
**Kamadjeu Raoul,** United States	**Kanmodi Kehinde,** Nigeria
**Karn Sandeep Kumar,** Nepal	**Kassogué Amadou,** Mali
**Kchir Hela,** Tunisia	**Kenni Audrey,** Cameroon
**Koui Tossea Stephane,** Cote D’ivoire	**Maghrebi Houcine,** Tunisia
**Mohamed Azhar Salim,** Comoros	**Mohammed Najat Kassim,** Tanzania
**Nardini Luisa,** South Africa	**Ndemwa Morris,** Kenya
**Ndounga Diakou Lee Aymar,** France	**Ngwa Moise,** United States
**Nounagnon Frutueux Agbangla,** France	**Oudjehih Messaouda,** Algeria
**Oyugi Elvis O,** Kenya	**Tachi Kenneth,** Ghana
**Tate Jacqueline E,** United States	**Touzani Rajae,** France
**Trigui Aymen,** Tunisia	**Waari Gabriel,** Kenya
**Wasswa Peter,** Uganda	**Zerouali Khalid,** Morocco

## Competing interests

The author is from the Pan African Medical Journal editorial office.

